# A Prospective, Active‐Controlled Split‐Face Clinical Trial Evaluating the Incremental Aesthetic Benefits of a Glutathione‐Containing Booster in a GHK‐Conjugated Spicule Regimen

**DOI:** 10.1111/jocd.71129

**Published:** 2026-08-25

**Authors:** Seongmin Noh, Wonkyu Hong, Hosung Choi, Youngjin Park, Do Young Rhee

**Affiliations:** ^1^ Department of Dermatology Benjamin Clinic Dermatology Seoul Republic of Korea; ^2^ Department of Dermatology Class One Clinic Seoul Republic of Korea; ^3^ Department of Dermatology LIEUL Seoul Clinic Seoul Republic of Korea; ^4^ Department of Dermatology Obliv Clinic Incheon Republic of Korea; ^5^ Department of Dermatology Heal House Dermatology Seoul Republic of Korea

## Abstract

**Background:**

Transdermal delivery of hydrophilic peptides such as GHK remains limited by the stratum corneum barrier and the classical 500 Da rule. Spicule‐based systems and redox‐responsive linkages have been investigated as strategies to enhance epidermal delivery, but in vivo glutathione‐triggered cleavage remains a proposed mechanism rather than a clinically proven process.

**Objectives:**

This study aimed to evaluate the clinical efficacy and safety of a GHK‐conjugated spicule regimen and to determine whether adding a multi‐ingredient, glutathione‐containing booster provides incremental aesthetic benefit compared with the shared base regimen alone.

**Methods:**

In this prospective, active‐controlled split‐face clinical trial, 20 healthy Asian women received five weekly facial treatments. One prespecified facial side received the base regimen consisting of a GHK‐conjugated spicule system and base concentrate, while the contralateral side received the same base regimen plus a separate multi‐ingredient booster containing 1% glutathione. Wrinkle depth, pore parameters, skin elasticity, dermal density, surface roughness, and pigmentation were evaluated at baseline, after one and three treatment sessions, and 4 weeks after the fifth session. Between‐side comparisons were performed using change‐from‐baseline values, with prespecified primary endpoints, multiplicity adjustment, and effect‐size reporting.

**Results:**

Both sides showed improvements from baseline. At the final timepoint, the intensive regimen produced greater improvements than the base regimen in periorbital wrinkle depth (−0.0144 vs. −0.0084 mm), nasolabial wrinkle depth (−0.0227 vs. −0.0173 mm), dermal density (+7.27 vs. +4.49), roughness (−1.23 vs. −0.73 μm), and mean pore volume (−0.0007 vs. −0.0005 mm^3^). Multiplicity‐adjusted between‐side differences remained significant for the prespecified primary endpoints and secondary endpoints. No adverse effects were reported.

**Conclusion:**

The GHK‐conjugated spicule base regimen improved multiple facial aging indicators, and the booster‐inclusive intensive regimen produced statistically greater short‐term improvements in this small split‐face trial. Because the booster was multi‐ingredient and the release mechanism was not directly measured in vivo, these findings should be interpreted as regimen‐level clinical evidence rather than proof of glutathione‐specific redox cleavage.

**Trial Registration:**

TRN Registration Number: PRE20250909‐005

## 1 Introduction

Peptides have become increasingly important in cosmetic dermatology due to their ability to modulate cellular activity, stimulate extracellular matrix synthesis, and promote skin regeneration. Among them, the tripeptide Gly‐His‐Lys (GHK) and its copper‐binding form GHK‐Cu have been widely investigated for their reparative and antiaging potential. Numerous studies have demonstrated that GHK peptides promote keratinocyte proliferation, support fibroblast function, regulate gene expression associated with wound repair, and enhance collagen and elastin production [1, 2, 3, 4]. GHK has additionally been reported to regulate genes associated with oxidative stress, inflammation, and tissue remodeling [5], to modulate multiple cellular pathways involved in skin regeneration [6], and to exert antioxidant and protective actions relevant to skin aging [7]. Despite these promising biological effects, the topical use of GHK peptides remains hampered by the formidable barrier posed by the stratum corneum, whose integrity is a principal determinant of percutaneous penetration [8]. According to the classical 500 Da rule, passive permeation across the intact stratum corneum is generally limited for compounds with molecular weights above ~500 Da [9]. However, even below this threshold, skin permeability depends strongly on physicochemical properties such as lipophilicity and charge; GHK is highly hydrophilic, which restricts its passive skin penetration. This constraint means that most topically applied peptides penetrate insufficiently to exert meaningful biological activity in vivo.

While chemical modifications such as oligoarginine‐mediated cell penetration have been proposed, these strategies do not adequately overcome the barrier imposed by the stratum corneum [10]. Consequently, there is a growing interest in physical and hybrid delivery strategies capable of enhancing peptide penetration without compromising safety or skin integrity.

Biogenic siliceous microneedles, derived from marine sponge spicules, have emerged as a novel transdermal delivery platform. Their rigid, needle‐like structure allows them to penetrate the superficial epidermis, creating thousands of transient microchannels that facilitate the passage of hydrophilic macromolecules. Previous studies have shown that combining spicules with extracellular vesicles, exosomes, or high–molecular‐weight polysaccharides significantly improves penetration and antiaging outcomes [11, 12, 13]. Importantly, spicules are composed of inert silica, remain temporarily embedded for up to 72 h, and are naturally expelled through epidermal turnover without biodegradation or inflammatory sequelae [14].

Sponge‐derived siliceous spicules have been characterized as natural microneedles for the transdermal delivery of hydrophilic biomacromolecules [15, 16]. Innovation in spicule‐based delivery has progressed from passive co‐application toward functionalization, in which bioactive molecules are conjugated directly to the spicule surface. The delivery system evaluated in this study uses GHK peptides covalently attached to the siliceous microneedles via a glutathione‐sensitive disulfide bond. In theory, once the spicules are inserted into the epidermis, the reducing environment from endogenous glutathione can cleave the bond to trigger localized peptide release. This redox‐responsive activation is a proposed mechanism based on preclinical research, rather than a proven in vivo outcome [17, 18, 19, 20]. Glutathione‐ and redox‐responsive linkers have been widely used to achieve triggered, site‐specific release in other delivery systems [21, 22], providing a conceptual basis for the disulfide chemistry employed here. This combination of mechanical insertion and redox‐triggered activation represents a hybrid delivery strategy with theoretical and preclinical advantages.

Supporting this concept, a preclinical ex vivo study found that spicule‐conjugated GHK penetrated skin dozens of times more effectively than a topical GHK application [23]. Likewise, in vivo tape‐stripping data (from treated human skin) [24], an established method for assessing stratum corneum penetration, showed higher peptide levels in the stratum corneum and epidermis when a GHK‐conjugated spicule system was used compared to a non‐spicule formulation. These findings, while promising, come from laboratory models and thus provide a basis for the current hypothesis but require clinical validation [25]. Despite these promising results, clinical evidence evaluating the performance of redox‐responsive spicule‐peptide conjugates remains limited.

The present study investigates the clinical efficacy of a GHK‐conjugated spicule system in human skin and further evaluates whether supplementing the treatment with an intensive multi‐ingredient booster containing glutathione and other active agents enhances outcomes. A split‐face design, an established approach for limiting interindividual variability in dermatologic studies [26], was adopted to evaluate the incremental effect of adding the booster to a shared active baseline. Accordingly, the present clinical trial was designed to evaluate the in vivo performance of the regimen, whereas the proposed mechanism of glutathione‐mediated disulfide cleavage remains speculative in the absence of direct in vivo measurement and is supported here only by preclinical and ex vivo data.

## 2 Methods

### 2.1 Study Design

This was a prospective, single‐center, investigator‐initiated, active‐controlled split‐face clinical trial. Each participant underwent five weekly treatment sessions. One facial side received the base (control) regimen, consisting of a GHK‐conjugated spicule system combined with a base concentrate containing baseline levels of glutathione. The contralateral side received the intensive (test) regimen, in which the same base regimen was supplemented with an additional, separate glutathione‐containing booster formulation. This design enabled evaluation of the incremental clinical benefit of the booster step added to a shared active baseline. Throughout the manuscript, the terms base/control and intensive/test are used consistently to avoid ambiguity.

Facial side (test vs. control) was assigned according to a prespecified, non‐randomized 1:1 allocation schedule managed by a staff member not otherwise involved in outcome assessment. Each enrolled participant received a sequential identifier and the corresponding prespecified side allocation, which was kept consistent across all visits to standardize the multistep protocol and minimize side mix‐ups during repeated sessions. Because non‐randomized side assignment can introduce lateral bias related to intrinsic facial asymmetry, sleep‐side preference, sun exposure, or baseline wrinkle distribution, baseline equivalence was assessed for all key outcomes and between‐side efficacy analyses were conducted on change‐from‐baseline values. Outcome assessors were blinded to treatment‐side allocation until completion of the statistical analysis; however, they were not blinded to visit timepoint, which is acknowledged as a limitation.

### 2.2 Participants

Twenty healthy Asian women between 30 and 65 years of age were enrolled. All participants exhibited visible signs of facial aging, including forehead lines, periorbital wrinkles, or decreased elasticity, consistent with characteristic intrinsic and extrinsic features of skin aging [27, 28]. Exclusion criteria included pregnancy or breastfeeding, active dermatologic disease, known ingredient hypersensitivity, recent aesthetic procedures, or use of topical agents likely to interfere with outcomes. All participants agreed to avoid introducing new skincare products during the study.

### 2.3 Preparation of Spicules and GHK Conjugates

Biogenic siliceous spicules were isolated from *Spongilla fragilis* using a multistep purification process involving sequential washing, alkaline and acid treatment, drying, grinding, and sieving. Following sterilization with ethylene oxide, the spicules were characterized by SEM and TEM to confirm structural integrity and surface uniformity.

To enable peptide conjugation, the purified spicules were functionalized with sulfhydryl groups using (3‐mercaptopropyl)trimethoxysilane. The sulfhydryl‐modified spicules were then reacted with HS‐GHK peptides under controlled pH conditions to form disulfide bonds. The resulting conjugates were washed thoroughly and dried. Representative spicule morphology exhibited lengths in the 150–250 μm and diameters in the 12–16 μm. These GHK‐conjugated spicules constitute the primary active ingredient in the cosmetic product tested (Peptaxel Intensive Solution Powder, SR Biotek).

### 2.4 Intervention Protocol

Prior to each session, the face was cleansed with a mild non‐exfoliating cleanser. The GHK‐conjugated spicule powder was freshly mixed with a standardized volume of the mixing ampoule of the commercial product (Regen Ampoule, SR Biotek) to ensure even dispersion. On the intensive‐regimen side, the glutathione‐containing booster was not mixed into the spicule formulation but was applied as a discrete subsequent step: after the spicule mixture had been massaged in, the booster was spread evenly over the side, covered with a sheet mask for 15 min, and the side was finished with a moisturizing cream. Because the booster constituted a separate step rather than a component of the spicule mixture, it did not modify the viscosity or dispersion of the spicule formulation or the spicule contact time on the intensive side.

The complete qualitative (INCI) compositions of all study formulations are provided in Table S1. The booster contained 1% glutathione; other concentration ratios are proprietary and were not disclosed by the manufacturer. In brief, both facial sides shared the same spicule powder and base concentrate, and the base concentrate already contained glutathione, whereas the intensive side additionally received a multi‐ingredient booster whose key actives include glutathione (1%), tranexamic acid, sodium hyaluronate, *Lactobacillus* extracellular vesicles, adenosine, copper tripeptide‐1 (GHK‐Cu), acetyl hexapeptide‐8, and sodium DNA. Because both sides shared this active baseline and the booster contributes several actives beyond glutathione, the incremental between‐side difference should be interpreted at the regimen level rather than as a glutathione‐specific effect.

Each formulation was massaged manually into the designated facial side for 10 min using standardized light pressure (< 200 g) and circular motions, following a consistent anatomical sequence beginning at the chin, progressing through the cheeks, and finishing at the forehead. This protocol ensured consistent intradermal insertion of spicules. A cooling step followed. Participants were instructed to avoid washing the face for at least 6 h. Because of the number of participants treated per session day, treatments were performed by three trained operators rather than a single operator (acknowledged as a limitation). Applied pressure was standardized by pretraining on a weighing scale and tactile calibration of the target force before treatment sessions. Posttreatment care was standardized by providing all participants the same barrier (“locking”) cream and sunscreen for use throughout the study, minimizing post‐care differences that could affect roughness, pigmentation, or ultrasound echogenicity.

### 2.5 Outcome Measures

Objective assessments were performed at baseline, after one and three treatment sessions, and 4 weeks after completing all five sessions.

- Baseline: before treatment
- T1: 1 week after the first treatment (following a single session)
- T2: 1 week after the third treatment (cumulative effect of three sessions)
- T3: 4 weeks after the fifth and final treatment (sustained effects post‐protocol)

The following noninvasive diagnostic tools were used:

- *Wrinkle depth* and *pore characteristics* were quantified using the Antera 3D imaging system. Surface roughness and melanin index (pigmentation) were additionally derived from the same Antera 3D imaging system.
- *Skin elasticity*, measured as the gravitational sagging angle, was evaluated using the F‐ray moiré imaging system.
- Skin density, defined as dermal echogenicity, was assessed using ultrasound‐based DUB SkinScanner imaging [29, 30].
- Safety and tolerability were monitored at every visit.

All Antera 3D, moiré, and ultrasound measurements were performed by the same trained operator under standardized room, lighting, and positioning conditions, minimizing inter‐operator variability. The operator was blinded to treatment‐side allocation but was not blinded to visit timepoint because the measurement schedule was sequential and recorded by visit. As these are region‐of‐interest‐based imaging devices, repeated‐measurement averaging was not performed in this clinical dataset; their repeatability has been documented previously (Antera 3D [31, 32]; ultrasound [33]), with high‐frequency ultrasound showing high intra‐observer reliability (ICC 0.946–0.978 for thickness and 0.648–0.865 for echogenicity [34]). As this was an efficacy rather than a device‐validation study, separate test–retest or intra‐operator ICC analyses were not performed. The device outputs are continuous metrics without universally accepted minimal clinically important difference thresholds; clinical relevance was therefore judged by absolute change from baseline, between‐side differences, responder analyses, and consistency with visual and subjective assessment [30, 31].

### 2.6 Statistical Analysis

Continuous variables were analyzed using repeated‐measures ANOVA or Friedman tests for within‐side changes over time, depending on distributional assumptions. Between‐side differences were assessed using paired *t*‐tests or Wilcoxon signed‐rank tests on change‐from‐baseline scores. Two primary endpoints were defined a priori for the final timepoint: nasolabial wrinkle depth and dermal density. The remaining parameters were treated as secondary endpoints. Within‐side changes were assessed with post hoc Bonferroni correction where applicable. Between‐side secondary endpoint analyses were adjusted using the Holm‐Bonferroni procedure to control the family‐wise error rate. Effect sizes (Cohen's *d*_*z*_ for paired data) and 95% confidence intervals are reported alongside *p* values. Statistical significance was defined as *p* < 0.05 after the applicable adjustment. All analyses were performed using SPSS.

### 2.7 Supportive Ex Vivo Penetration and Cleavage Assay

**FIGURE 1:**
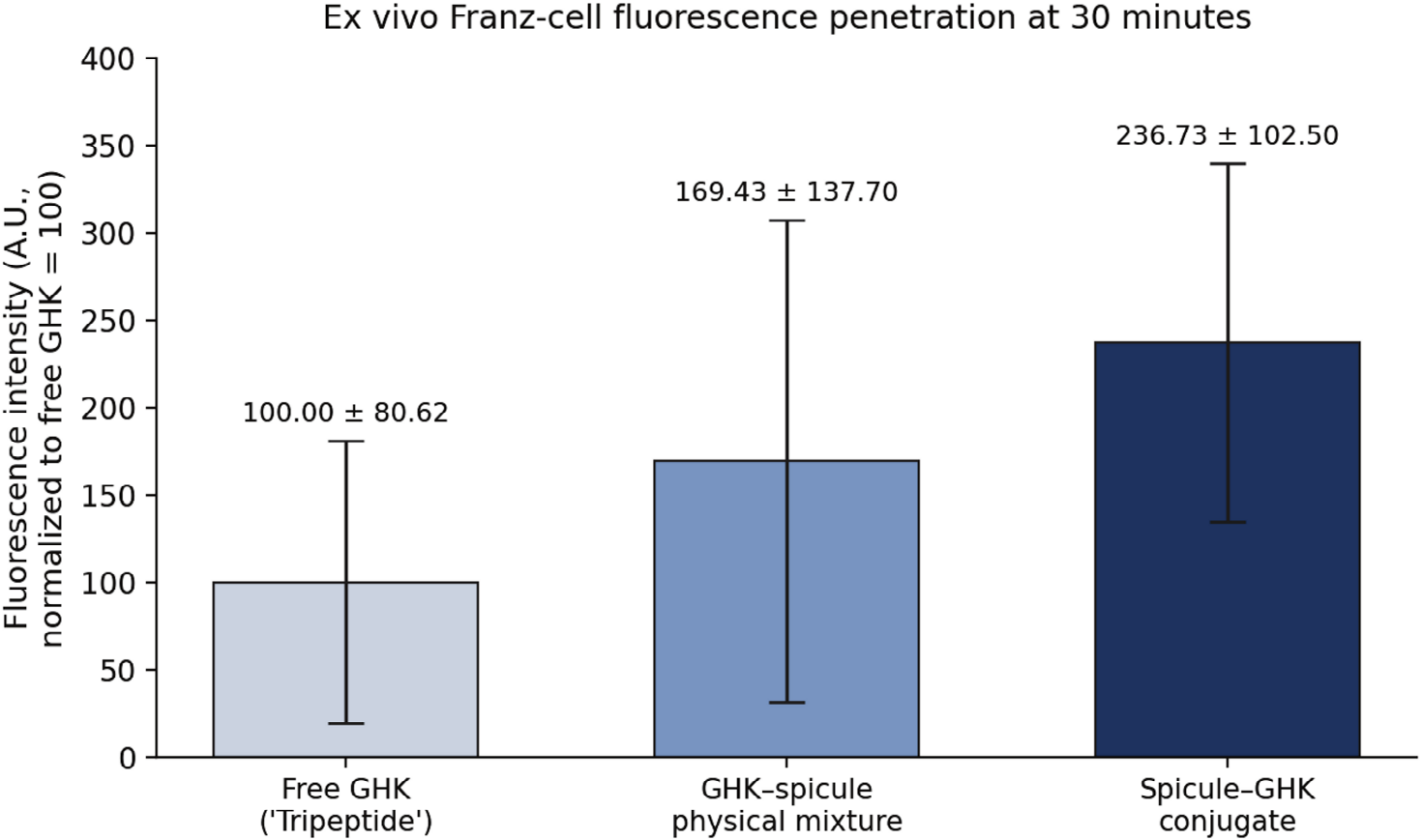
Ex vivo Franz diffusion‐cell fluorescence penetration of free GHK (“Tripeptide”), an unconjugated GHK–spicule physical mixture, and the spicule–GHK conjugate at 30 min. Fluorescence intensity is expressed in arbitrary units (A.U.) normalized to free GHK (= 100). Data are mean ± SD (*n* = 3).

The fluorescence penetration data shown in Figure 1 were obtained from a supportive ex vivo Franz diffusion‐cell assay [35] and were not part of the clinical trial efficacy dataset. Free GHK (“Tripeptide”), an unconjugated GHK‐spicule physical mixture, and the spicule‐GHK conjugate were applied to the skin model under identical exposure conditions. Fluorescence intensity was measured at the indicated timepoint and reported in arbitrary units (A.U.) normalized to free GHK. Reductive cleavage was assessed separately under DTT conditions (0.4–0.6 M) as a laboratory surrogate for a reducing glutathione‐rich environment. These data were used only to support biological plausibility and were not used for clinical endpoint testing or multiplicity‐adjusted clinical analyses.

## 3 Results

### 3.1 Participant Characteristics

All 20 participants completed the protocol. The mean age was 55.20 ± 8.22 years. No participants reported lifestyle or skincare changes during the study. No adverse reactions occurred.

### 3.2 Pore Parameters

**FIGURE 2:**
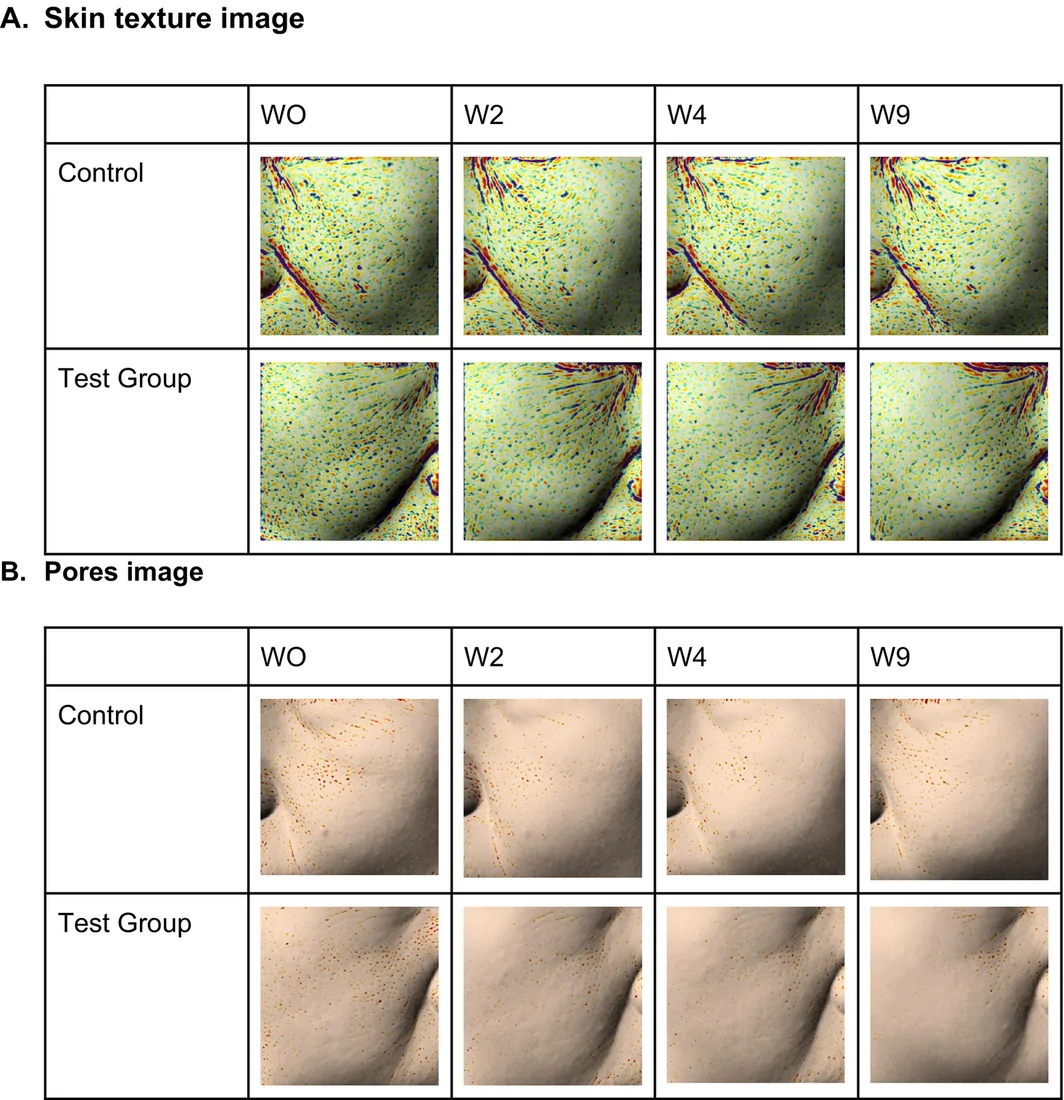
Skin texture and pores images at baseline(W0), week 2(W2), week 4(W4), and week 9(W9) for the control and test (booster‐treated) sides. (a) Skin texture images. (b) Pore images.

Quantitative analysis of pore volume and surface roughness revealed significant aesthetic improvements in both groups, with the booster‐treated side again achieving superior results. The roughness average (Ra) and mean pore volume consistently declined with ongoing treatments. While both facial sides demonstrated improvements, the booster‐treated side showed a slightly greater reduction in pore volume at the final timepoint, though not all differences reached statistical significance. These findings suggest that structural remodeling produced by GHK activity is achieved primarily through the base spicule‐peptide system, with modest enhancement from the booster (Table 1 and Figure 2).

**TABLE 1:** Changes in pore parameters over time.

Parameter		Control	Test	*p* value between the groups
Mean pore volume (mm^3^)	Baseline	0.0021 ± 0.0007	0.0021 ± 0.0006	
	T1	0.0019 ± 0.0006	0.0018 ± 0.0006	0.002
	T2	0.0017 ± 0.0006	0.0015 ± 0.0005	< 0.001
	T3	0.0016 ± 0.0005	0.0014 ± 0.0004	< 0.001
	*p* value between T3 and baseline	< 0.001^	< 0.001	
Ra (μm)	Baseline	7.91 ± 1.46	7.93 ± 1.43	
	T1	7.59 ± 1.38	7.38 ± 1.37	0.021
	T2	7.38 ± 1.33	6.98 ± 1.36	0.001
	T3	7.18 ± 1.31	6.70 ± 1.35	< 0.001
	*p* value between T3 and baseline	< 0.001	< 0.001	

*Note:* ∧ Friedman test used for within‐side comparison (non‐normal distribution); repeated‐measures ANOVA used for all unmarked within‐side *p*‐values.

### 3.3 Skin Elasticity

**FIGURE 3:**
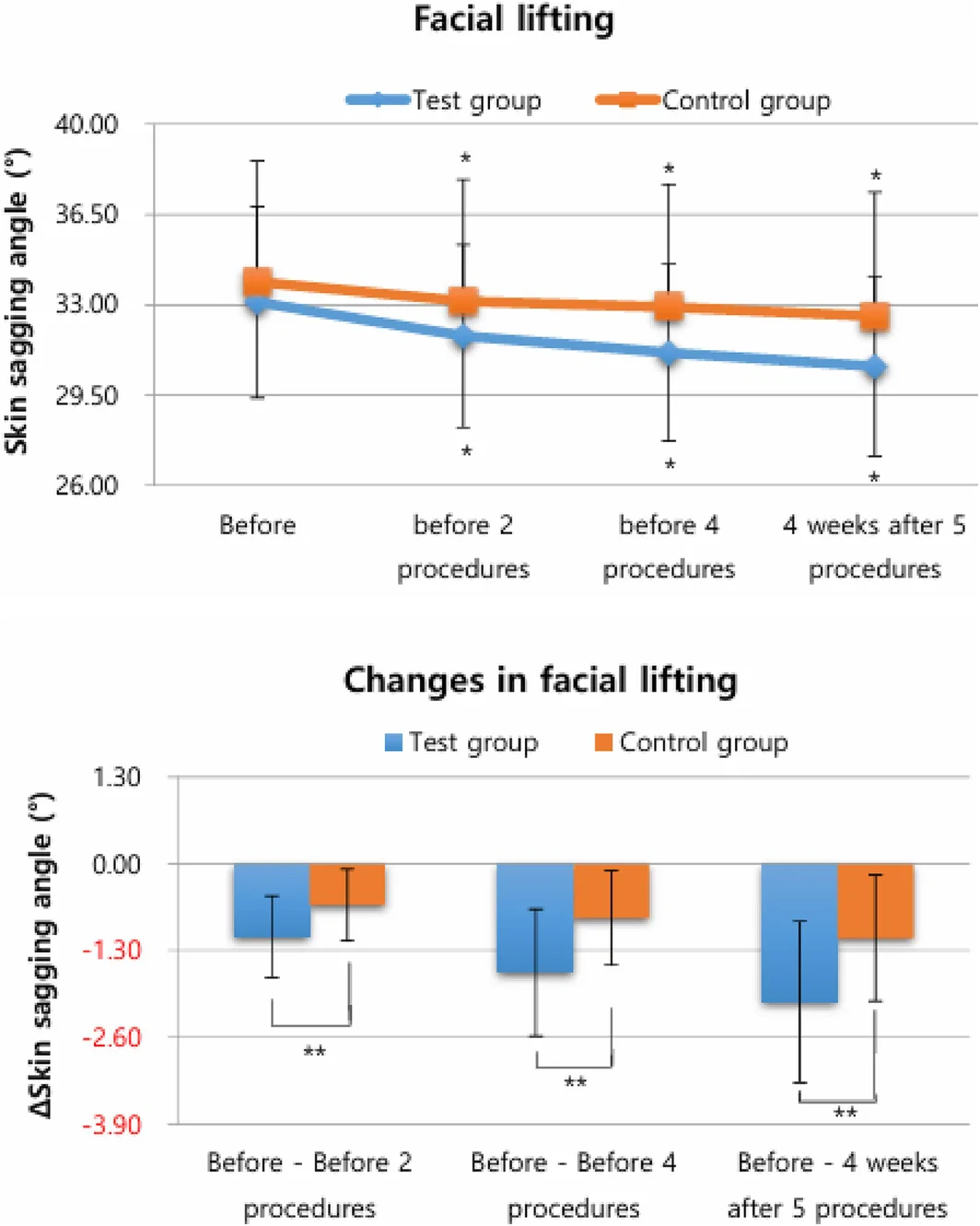
Sagging angle comparison between the base (control) and intensive (test) sides. *p < 0.05 for within‐group change from baseline (repeated‐measures ANOVA/Friedman). **p < 0.05 for between‐group (test vs. control) difference at that timepoint (paired t‐test/Wilcoxon signed‐rank).

Skin elasticity, assessed by the sagging angle, improved significantly on both sides of the face after two treatments, with further improvement after four treatments and sustained gains at the final follow‐up. The magnitude of improvement was greater on the booster‐treated side, achieving statistical significance at the late timepoints (Table 2 and Figure 3).

**TABLE 2:** Changes in skin elasticity (sagging angle).

Parameter		Control	Test	*p* value between the groups
Skin elasticity	Baseline	33.88 ± 4.69	33.10 ± 3.71	
	T1	33.14 ± 4.71	31.79 ± 3.53	< 0.001
	T2	32.91 ± 4.75	31.14 ± 3.43	< 0.001
	T3	32.55 ± 4.80	30.61 ± 3.47	< 0.001
	*p* value between T3 and baseline	< 0.001	< 0.001	

### 3.4 Skin Density

**FIGURE 4:**
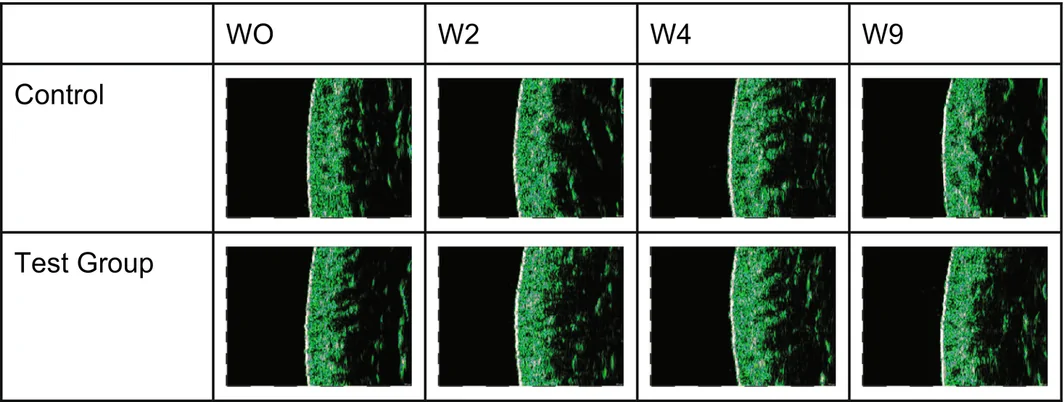
Dermal ultrasound echogenicity images.

Dermal echogenicity, a critical indicator of skin firmness and collagen presence, increased steadily across all evaluation points on both sides of the face, indicating progressive enhancement of dermal structural integrity. Although increases were observed bilaterally, the booster‐treated side demonstrated a more pronounced elevation in dermal density during the final assessment, consistent with enhanced ECM remodeling (Table 3 and Figure 4).

**TABLE 3:** Changes in dermal echogenicity (skin density).

Parameter		Control	Test	*p* value between the groups
Dermal density	Baseline	58.49 ± 4.18	57.57 ± 4.20	
	T1	60.35 ± 4.68	61.44 ± 4.48	0.003
	T2	61.86 ± 5.00	63.68 ± 5.04	< 0.001
	T3	62.98 ± 3.96	64.84 ± 4.28	< 0.001
	*p* value between T3 and baseline	< 0.001	< 0.001	

### 3.5 Wrinkle Depth

**FIGURE 5:**
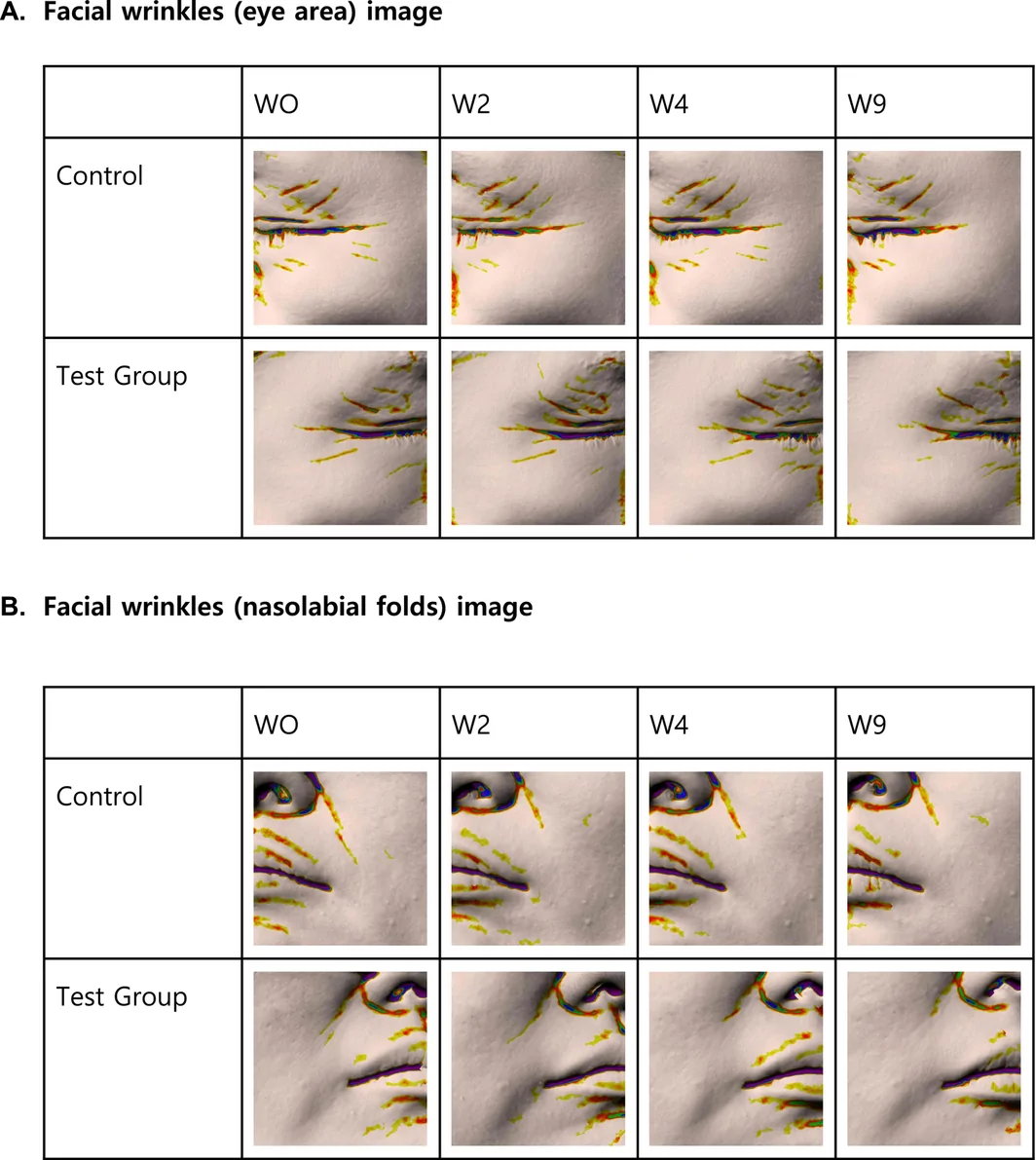
Representative Antera 3D wrinkle images at baseline(W0), week 2(W2), week 4(W4), and week 9(W9) for the control and test (booster‐treated) sides. (A) Facial wrinkles (periorbital/eye area). (B) Facial wrinkles (nasolabial folds).

Periorbital and nasolabial wrinkle depth decreased significantly relative to baseline following two treatments and continued to decline through the posttreatment follow‐up. Notably, the booster‐treated side exhibited more substantial reductions in nasolabial wrinkle depth at the final two assessments, reaching statistical significance (Table 4 and Figure 5).

**TABLE 4:** Changes in wrinkle depth.

Parameter		Control	Test	*p* value between the groups
Nasolabial fold, average depth (mm)	Baseline	0.0931 ± 0.0182	0.0930 ± 0.0203	
	T1	0.0837 ± 0.0153	0.0805 ± 0.017	0.037
	T2	0.0802 ± 0.0142	0.0753 ± 0.0154	0.002
	T3	0.0759 ± 0.0145	0.0703 ± 0.0142	0.016
	*p* value between T3 and baseline	< 0.001^	< 0.001^	
Periorbital wrinkles, average depth (mm)	Baseline	0.0938 ± 0.0158	0.0935 ± 0.0194	
	T1	0.0896 ± 0.0145	0.0861 ± 0.0170	0.007
	T2	0.0875 ± 0.0135	0.0839 ± 0.0165	0.025
	T3	0.0854 ± 0.0122	0.0791 ± 0.0136	0.006
	*p* value between T3 and baseline	< 0.001^	< 0.001	

*Note:* ∧ Friedman test used for within‐side comparison (non‐normal distribution); repeated‐measures ANOVA used for all unmarked within‐side *p*‐values.

Between‐side incremental analysis. Baseline values did not differ significantly between the two sides for the key prespecified outcomes (all *p* > 0.05), supporting baseline comparability despite the absence of left–right randomization. At the final timepoint, the intensive (booster) side showed significantly greater improvement than the base side for both prespecified primary endpoints (nasolabial wrinkle depth and dermal density) and for the secondary endpoints summarized in Table 5. The between‐side difference in change from baseline, effect size (Cohen's *d*_*z*_), 95% CI, and Holm‐adjusted *p* value for each endpoint are presented in Table 5.

**TABLE 5:** Between‐side incremental effect at the final timepoint (4 weeks after the fifth session; *n* = 20). Values are the paired difference in change from baseline (intensive − base side); negative values indicate greater improvement on the intensive side for wrinkle depth, sagging angle, melanin, roughness, and pore volume, and the positive value indicates greater density gain. *p* values are Holm–Bonferroni‐adjusted.

Endpoint	Between‐side Δ (change from baseline)	95% CI	Cohen's *d*_*z*_	Holm‐adj. *p*
Nasolabial wrinkle depth (primary)	−0.0054 mm	−0.0097 to −0.0011	−0.59	0.016
Dermal density (primary)	+2.78	+1.73 to +3.82	+1.25	< 0.001
Periorbital wrinkle depth	−0.0060 mm	−0.0100 to −0.0019	−0.69	0.012
Skin sagging angle	−1.15°	−1.62 to −0.68	−1.14	< 0.001
Melanin index	−1.19	−1.67 to −0.71	−1.16	< 0.001
Roughness	−0.50 μm	−0.69 to −0.30	−1.19	< 0.001
Mean pore volume	−0.0002 mm^3^	−0.0003 to −0.0001	−0.90	0.002

*Note:* *d*_*z*_ = standardized paired mean difference (Cohen's *d* for paired data). Two primary endpoints (nasolabial wrinkle depth and dermal density) were defined a priori; the remaining endpoints were secondary and corrected for multiplicity using the Holm–Bonferroni procedure.

To convey absolute magnitude alongside relative change, mean absolute changes from baseline (with percentages) and responder rates by side at the final timepoint are summarized in Table 6. These values show that the intensive (booster) side produced numerically greater mean changes and higher responder rates than the base side across the reported parameters, although the modest absolute magnitude of several outcomes should be interpreted cautiously.

**TABLE 6:** Absolute and relative change from baseline by facial side at the final timepoint (4 weeks after the fifth session; *n* = 20), with responder rates.

Parameter	Base (control) side	Intensive (test) side	Responders, intensive vs. base
	Mean change (improvement %)	Mean change (improvement %)	
Periorbital wrinkle depth	−0.0084 mm (9.0%)	−0.0144 mm (15.4%)	70% vs. 35% (≥ 10% reduction)
Nasolabial wrinkle depth	−0.0173 mm (18.5%)	−0.0227 mm (24.4%)	65% vs. 35% (≥ 20% reduction)
Dermal density	+4.49 (7.7%)	+7.27 (12.6%)	60% vs. 25% (≥ 10% increase)
Roughness	−0.73 μm (9.2%)	−1.23 μm (15.5%)	65% vs. 5% (≥ 15% reduction)
Mean pore volume	−0.0005 mm^3^ (23.8%)	−0.0007 mm^3^ (33.3%)	—

*Note:* Values are mean change from baseline; improvement percentage is shown in parentheses (reduction for wrinkle depth, roughness, and pore volume; increase for dermal density). Responders are the proportion of sides meeting the prespecified improvement threshold shown for each parameter. A pore‐volume responder threshold was not prespecified (—).

### 3.6 Assessment of Improvement of Subject Satisfaction

According to posttreatment surveys using a non‐validated internal questionnaire, over 90% of participants reported noticeable improvements in skin texture, clarity, and tightness on the intensive side. These satisfaction findings were considered supportive only and were not treated as confirmatory efficacy endpoints.

No adverse skin reactions were observed based on subject questionnaires throughout the study period. Evaluated parameters included erythema, swelling, itching, pain, and other symptoms, all of which scored zero at every time point. No treatment or compensation was required.

## 4 Discussion

This clinical trial provides evidence that a GHK‐conjugated spicule delivery system improves several key skin‐aging parameters, including wrinkles, pores, elasticity, and dermal density. The split‐face design enabled a direct within‐subject comparison between the base regimen and an intensive regimen supplemented with a multi‐ingredient, glutathione‐containing booster.

The observed improvements are consistent with the established biological effects of GHK peptides, including pro‐repair signaling and extracellular matrix remodeling. Dermal ultrasound findings further supported these effects by demonstrating increased echogenic density, reflecting improved dermal compactness and collagen fiber organization. The use of high‐frequency ultrasound for dermatologic evaluation is increasingly supported in clinical dermatology; recent systematic reviews of FDA‐ and EMA‐approved noninvasive imaging modalities for basal cell carcinoma include high‐frequency ultrasound among evaluated/approved dermatologic imaging techniques [36, 37].

Overall outcomes may be explained by a dual‐step delivery concept. First, biogenic spicules mechanically bypass the stratum corneum by generating transient microchannels. Second, the glutathione‐responsive disulfide linkage is designed to allow peptide release in a reductive cutaneous environment. This interpretation is supported by preclinical Franz cell diffusion assays, in which the spicule‐GHK conjugate achieved higher penetration (236.73 ± 102.50 A.U. at 30 min) than free GHK (100.00 ± 80.62 A.U.) and an unconjugated physical mixture of GHK and spicules (169.43 ± 137.70 A.U.). In this assay, penetration was quantified by fluorescence in arbitrary units (A.U.), normalized to free GHK (referenced to 100); in Figure 1, “Tripeptide” denotes free, unconjugated GHK. These Franz‐cell and cleavage data are preclinical, ex vivo findings and are presented as supportive mechanistic evidence rather than as part of the clinical trial dataset. In complementary ex vivo experiments, reducing conditions (0.4–0.6 M DTT, used as a reductive surrogate for glutathione) induced rapid cleavage of GHK from the spicules, supporting the feasibility of redox‐triggered release under laboratory conditions.

Clinically, the booster‐supplemented regimen produced greater improvements in parameters such as elasticity, dermal density, and wrinkle depth, suggesting incremental benefit when added to a shared active baseline. Because this booster is a multi‐ingredient formulation and because both sides contained active ingredients, the observed incremental effect should be interpreted at the regimen level, consistent with common real‐world approaches that use combined actives to optimize aesthetic outcomes.

Taken together, although the ex vivo findings provide a plausible biochemical rationale, the in vivo release mechanism was not directly quantified in participants. In addition, glutathione was present at baseline in the control vehicle, and both regimens contain multiple bioactive components; therefore, the between‐side differences should be interpreted as the cumulative clinical benefit of the intensive regimen rather than definitive causal evidence of glutathione‐mediated cleavage. Potential side‐related confounding (e.g., intrinsic facial biomechanical asymmetry, sleep‐side preference, uneven sun exposure, and excipient‐dependent penetration effects) may also have contributed to the observed differences. Moreover, although several between‐side differences were statistically significant and supported by moderate‐to‐large effect sizes, the absolute magnitudes of change were modest; their clinical relevance should therefore be interpreted cautiously and confirmed in larger, longer studies with patient‐reported outcomes and blinded global aesthetic assessments.

Importantly, the regimen was well tolerated, with no adverse events. Future studies incorporating a fully randomized, double‐blind split‐face design and factorial arms (e.g., glutathione‐free base and glutathione‐only booster conditions) would enable clearer attribution of redox‐specific contributions while further validating delivery‐driven efficacy.

## 5 Limitations

While the results are promising, the study is limited by its short follow‐up period and relatively small sample size (*n* = 20), which limits generalizability. Side allocation was not randomized between the two facial sides; although assessors were blinded to side allocation and baseline equivalence was assessed, this non‐randomized, single‐blind, split‐face design cannot fully exclude residual lateral bias arising from intrinsic facial asymmetry, sleep‐side preference, or uneven environmental exposures. Treatments were performed by three trained operators rather than a single operator, although pressure and sequence were standardized. Efficacy was assessed mainly through instrument‐based surrogate measures; no blinded Global Aesthetic Improvement Scale (GAIS) grading or validated patient‐reported outcome (PRO) instrument was used, and the subject‐satisfaction data were obtained with a non‐validated internal questionnaire. Further research should evaluate durability across broader patient populations and incorporate fully randomized, double‐blind, factorial designs to isolate the contribution of glutathione from other booster components and to reduce spatial and subjective biases.

## 6 Conclusion

The GHK‐conjugated spicule base regimen effectively ameliorated multiple signs of facial aging, and the booster‐inclusive intensive regimen yielded statistically greater short‐term improvements in several parameters compared with the base regimen. However, this small, short‐term, non‐randomized split‐face study supports the intensified regimen only at the regimen level; larger randomized, double‐blind, and factorial studies are needed to confirm durability, establish clinical meaningfulness, and isolate the contribution of glutathione from that of other booster components.

## Author Contributions

S.N., W.H., and H.C. performed the research. S.N., W.H., H.C., and D.Y.R. designed the research study. Y.P. and D.Y.R. contributed essential reagents or tools. S.N. and W.H. analyzed the data. S.N. and H.C. wrote the paper. All authors have read and approved the final manuscript.

## Ethics Statement

Approved by the Institutional Review Board of Human Co. Ltd. Skin Clinical Trial Center (IRB No. HM‐IRB‐P24‐0513).

## Consent

All participants provided written informed consent.

## Conflicts of Interest

The authors declare no conflicts of interest.

## Supporting information


**Table S1:** Full qualitative compositions (INCI) of the study formulations.

## Data Availability

The data that support the findings of this study are available on request from the corresponding author. The data are not publicly available due to privacy or ethical restrictions.
