# Supplementary material for: A Prospective, Active‐Controlled Split‐Face Clinical Trial Evaluating the Incremental Aesthetic Benefits of a Glutathione‐Containing Booster in a GHK‐Conjugated Spicule Regimen
Source: J Cosmet Dermatol. 2026 Aug 25;25(9):e71129. doi: 10.1111/jocd.71129 (PMC13504699; doi:10.1111/jocd.71129)
Supplement: Supplementary file 1 [file JOCD-25-e71129-s001.docx]

# Supplementary Table S1.

**Full qualitative compositions (INCI) of the study formulations.**

Ingredients are listed in descending order of content as declared by the manufacturer. Concentration ratios are proprietary and are not disclosed. Both facial sides received the spicule powder and the base concentrate; the intensive (test) side additionally received the booster.

| Formulation (role) | Ingredients (INCI) |
| --- | --- |
| Spicule powder (Step 1; both sides) | Betaine, Silica Polysilsesquioxane Propyldisulfideacetyl Tripeptide-84, Hydrolyzed Sponge, Dipotassium Glycyrrhizate, Biotin, Niacinamide, Adenosine |
| Base concentrate / mixing ampoule (Step 2; both sides) | Water, Butylene Glycol, 1,2-Hexanediol, Glycerin, Niacinamide, C12-14 Alketh-12, Carbomer, Tromethamine, Ethylhexylglycerin, Sodium DNA, Adenosine, Glutathione, Glyceryl Glucoside, Sodium Hyaluronate, Pentylene Glycol, Ectoin, Caprylhydroxamic Acid, Glycine, Paeonia Suffruticosa Root Extract, Prunus Mume Fruit Extract, Chrysanthemum Zawadskii Extract, Serine, Glutamic Acid, Aspartic Acid, Leucine, Vincetoxicum Atratum Extract, Tocopherol, Alanine, Lysine, Arginine, Tyrosine, Phenylalanine, Valine, Threonine, Proline, Isoleucine, Histidine, Methionine, Cysteine |
| Booster (intensive/test side only) | Water, Glutathione, Tranexamic Acid, Sodium Chloride, Sodium Hyaluronate, Sodium Hydroxide, Lactobacillus Extracellular Vesicles, Adenosine, Disodium Phosphate, Potassium Chloride, Pyridoxine HCl, Copper Tripeptide-1, Acetyl Hexapeptide-8, Sodium DNA, Potassium Phosphate, Dipotassium Phosphate |
